# Affirmative Action Repeal and Racial and Ethnic Diversity in US Medical School Admissions

**DOI:** 10.1001/jamanetworkopen.2025.35020

**Published:** 2025-10-10

**Authors:** Natalie Florescu, Andrea Lin, Samantha Temucin, Lisa Rae

**Affiliations:** 1 Lewis Katz School of Medicine at Temple University, Philadelphia, Pennsylvania; 2 Department of Surgery, University of Vermont Medical Center, Burlington, Vermont; 3 Philadelphia College of Osteopathic Medicine, Philadelphia, Pennsylvania

## Abstract

**Importance:**

In June 2023, the US Supreme Court’s decision in *Students for Fair Admissions v. Harvard University* effectively overturned affirmative action, raising concerns about the future of diversity in medical education. A diverse physician workforce is crucial for advancing health care equity and improving patient outcomes.

**Objective:**

To evaluate the association between the Supreme Court’s ruling and the racial and ethnic composition of students matriculating into Doctor of Medicine (MD) and Doctor of Osteopathic Medicine (DO) programs.

**Design, Setting, and Participants:**

Cross-sectional analysis of comprehensive publicly available data on medical school applications and matriculation by race and ethnicity from 2020 to 2024. Data were obtained from the Association of American Medical Colleges (AAMC) and the American Association of Colleges of Osteopathic Medicine (AACOM) to compare across MD and DO programs. Five application cycles were analyzed, with 4 representing preruling trends.

**Main Outcome and Measure:**

Change in the racial and ethnic composition of matriculating medical students following the *Students for Fair Admissions v. Harvard University* decision.

**Results:**

From 2020 to 2025, there were a mean (SD) of 55 037 (4315) applicants and 22 750 (349) matriculants to MD institutions and 214 163 (17 243) applicants and 9019 (274) matriculants to DO institutions. Significant disparities in application and matriculation patterns were observed across both program types, with persistent underrepresentation among American Indian or Alaska Native, Black, Hispanic, Native Hawaiian, and multiracial applicants. Between the 2023 to 2024 and 2024 to 2025 academic years, MD matriculation declined by 11.6% for Black students (χ^2^_1_ = 40.59; *P* < .001) and 10.8% for Hispanic students (χ^2^_1_ = 45.87; *P* < .001), respectively. DO programs showed broader underrepresentation. Compared with White and Asian applicants, all other racial groups were significantly underrepresented in both applications and matriculation.

**Conclusions and Relevance:**

These declines threaten progress toward health care equity. Medical schools must explore alternative admissions strategies, such as holistic review processes that account for structural barriers. Without such efforts, reduced diversity in medical education may worsen existing health disparities.

## Key Points

**Question:**

Was the US Supreme Court decision ending affirmative action associated with changes in the racial and ethnic composition of medical school matriculants?

**Findings:**

In this cross-sectional analysis of Association of American Medical Colleges and American Association of Colleges of Osteopathic Medicine data including a mean of 55 037 applicants to Doctor of Medicine (MD) programs and 214 163 applicants to Doctor of Osteopathic Medicine (DO) programs from 2020 to 2024, significant declines in MD matriculation were observed between 2023 and 2024 among Black (−11.6%) and Hispanic/Latinx (−10.8%) students, while matriculation of Asian and White students increased. DO programs showed persistent underrepresentation of all underrepresented in medicine groups.

**Meaning:**

These findings suggest that elimination of race-conscious admissions coincided with declines in medical school diversity, threatening progress toward health care equity and highlighting the need for alternative strategies to promote representation.

## Teaser

This cross-sectional study evaluates the association between the US Supreme Court’s *Students for Fair Admissions v. Harvard University* ruling and the racial and ethnic composition of students matriculating into Doctor of Medicine and Doctor of Osteopathic Medicine programs.

## Introduction

In June 2023, the US Supreme Court’s decision in *Students for Fair Admissions v. Harvard* effectively overturned affirmative action, ruling that race-conscious admission policies violated the Equal Protection Clause of the Fourteenth Amendment.^1^ In the wake of this decision, the undergraduate class of 2028 at several universities showed a decline in racial diversity compared with previous years, with Harvard’s percentage of Black students dropping by 4%.^2^ State-based bans on affirmative action, beginning in the 1990s, have been associated with decreased racial diversity in both undergraduate and graduate institutions in those states.^3,4^ Despite these challenges, there has been a sustained push for greater diversity in medical education, driven by the well-documented benefits of diverse health care teams on patient care.^5,6,7,8,9,10^

The Association of American Medical Colleges (AAMC) defines underrepresented in medicine (URIM) as those who self-identify as American Indian or Alaska Native, Black or African American, Hispanic or Latino, or Native Hawaiian or Other Pacific Islander.^11^ Over the last 10 years, AAMC matriculation data from Doctor of Medicine (MD) programs showed steady increases among rates of URIM applicants and matriculants, where 19.4% of students identified as URIM before the 2023 Supreme Court ruling.^12,13^ Data from the American Association of Colleges of Osteopathic Medicine (AACOM) showed that only 11.2% of students in Doctor of Osteopathic Medicine (DO) programs identified as URIM.^14^ According to 2023 US Census data, approximately 35% of the US population identifies as these same racial groups,^15^ demonstrating that neither MD nor DO programs have achieved a representative level of racial diversity. Morris et al^16^ analyzed MD matriculation data from 1978 to 2019 and found that URIM groups remained significantly underrepresented compared with their proportions in the 2019 census. This study aims to examine the association between the 2023 Supreme Court ruling and the demographics of matriculating medical students at both DO and MD institutions, as we now face the possibility that the hard-earned progress toward diversity may be undermined.

## Methods

AAMC and AACOM collect demographic data on all MD and DO applicants as part of their online application systems, the American Medical College Application Service (AMCAS) and the American Association of Colleges of Osteopathic Medicine Application Service (AACOMAS), respectively. Additionally, all accredited MD and DO institutions report demographic information of matriculating students to AAMC or AACOM annually. AAMC aggregates institutions’ data and publishes it publicly.^12,13^ AACOM publishes data on applicants and matriculants, separated by institution.^14^ Both AAMC and AACOM report race and ethnicity using the following groupings: American Indian or Alaska Native, Asian, Black or African American, Hispanic or of Spanish Origin, Native Hawaiian or Other Pacific Islander, and White. Because this study was limited to the analysis of publicly available and fully anonymized data, this study qualified as exempt from institutional review board oversight at Temple University. This study followed the Strengthening the Reporting of Observational Studies in Epidemiology (STROBE) reporting guidelines.

### Statistical Analysis

In March of 2025, publicly available race and ethnicity data in medical school applications and matriculations were taken from both associations from 2020 to 2024. A 5-year timeframe was selected because the AAMC’s publicly available racial demographic data begin in 2020. For each year, the proportion of applicants and matriculants by race was calculated using the raw data. Using SPSS version 29 (IBM), statistical analysis was performed to compare annual application and matriculation proportions within each race and ethnicity group to compare the proportions of application and matriculation of URIM groups with those considered represented in medicine (White and Asian), to evaluate the trends in proportions of each racial and ethnic group among applicants and matriculants over time, and to assess the differences in matriculation proportions between the 2023 and 2024 entering classes. Paired *t* tests, independent *t* tests, linear regression, and χ^2^ tests were performed where appropriate.

## Results

Across both MD and DO programs, significant racial disparities existed in medical school application and matriculation patterns from 2020 to 2024. Notably, the results suggest both consistent underrepresentation of certain racial groups and shifting trends that coincide with the end of affirmative action.

Over the last 5 cycles, Asian (mean [SD] applied, 0.266 [0.019]; matriculated, 0.282 [0.026]; *t*_4_ = 4.683; 95% CI, −0.026 to −0.007; *P* = .01) and White (mean [SD] applied, 0.497 [0.014]; matriculated, 0.517 [0.010]; *t*_4_ = 7.113; 95% CI, −0.028 to −0.012; *P* = .001) applicants to MD programs had significantly higher matriculation rates than application rates, while Black applicants had significantly lower matriculation rates than application rates (mean [SD] applied, 0.108 [0.007]; matriculated, 0.099 [0.009]; *t*_4_ = 2.250; 95% CI, −0.002 to −0.010; *P* = .04). Although differences were not statistically significant for American Indian or Alaska Native, Hispanic, and Native Hawaiian groups, their application and matriculation numbers remain disproportionately low (Table 1). Comparing 2023 and 2024 data, MD programs saw significant increases in matriculation among Asian (χ^2^_1_ = 12.484; *P* < .001) and White (χ^2^_1_ = 27.55; *P* < .001) individuals, while matriculation among Black (χ^2^_1_ = 39.62; *P* < .001) and Hispanic (χ^2^_1_ = 45.581; *P* < .001) individuals declined significantly. Despite stable application proportions, matriculation for Black and Hispanic individuals experienced relative decreases of 11.6% (2304 students in 2023 vs 2036 students in 2024) and 10.8% (2910 students in 2023 vs 2595 students in 2024), respectively. Matriculation of American Indian or Alaska Native students also relatively decreased by 22.1%; however, this finding was not statistically significant.

**Table 1.** Racial Composition of Doctor of Medicine Applicant Pool and Matriculated Students^a^

Race and Ethnicity	Participants, No. (%)									
	2020		2021		2022		2023		2024	
	Applied	Matriculated	Applied	Matriculated	Applied	Matriculated	Applied	Matriculated	Applied	Matriculated
American Indian or Alaska Native	561 (1.1)	248 (1.1)	689 (1.1)	227 (1.0)	563 (1.0)	225 (1.0)	583 (1.1)	258 (1.1)	493 (0.9)	201 (0.8)
Asian	13 018 (24.5)	5543 (24.9)	15 588 (25.0)	6004 (26.5)	14 752 (26.7)	6524 (28.7)	14 553 (27.7)	6758 (29.4)	15 101 (29.1)	7323 (31.6)
Black or African American	5197 (9.8)	2117 (9.5)	7331 (11.7)	2562 (11.3)	5922 (10.7)	2308 (10.1)	5665 (10.8)	2304 (10.0)	5824 (11.2)	2036 (8.8)
Hispanic, Latino, or of Spanish origin	5820 (11.0)	2678 (12.0)	7281 (11.7)	2869 (12.6)	6247 (11.3)	2784 (12.2)	6107 (11.6)	2910 (12.6)	6239 (12.0)	2595 (11.2)
Native Hawaiian or other Pacific Islander	214 (0.4)	80 (0.3)	256 (0.4)	85 (0.4)	222 (0.4)	101 (0.4)	246 (0.5)	94 (0.4)	246 (0.5)	90 (0.4)
White	27 235 (51.4)	11 874 (53.4)	31 028 (49.7)	11 682 (51.5)	27 818 (50.4)	11 800 (52.0)	26 020 (49.5)	11 775 (51.2)	24 668 (47.5)	11 738 (50.7)
URIM, %^b^	22.3	22.9	24.9	25.3	23.4	23.7	24.0	24.1	24.6	21.2
Total No.^c^	53 030	22 239	62 443	22 666	55 189	22 710	52 577	22 980	51 946	23 156

Abbreviation: URIM, underrepresented in medicine.

^a^ Applicant and matriculant data were calculated using raw data presented by the AAMC in Tables A-14.1 and A-14.3. Race and ethnicity categories reflect responses reported as “alone or in combination,” such that individuals may be represented in more than 1 category. Because duplicate individuals across categories cannot be identified, analyses are based on non–mutually exclusive racial groupings. Individuals reporting other or unknown race were not included here.

^b^ URIM data presented here were calculated using those who identified as American Indian or Alaska Native; Black or African American; Hispanic, Latino, or of Spanish origin; and Native Hawaiian or other Pacific Islander.

^c^ Totals listed here are taken directly from the AAMC’s total unduplicated applicant list, and given that individuals who selected other or unknown race were not included in this study, and individuals could select more than 1 response, the data in each column add to the total unduplicated applicant list.

DO programs revealed a broader statistically significant pattern of under-matriculation across all URIM groups: the proportion of matriculation was lower than the proportion of applications for American Indian or Alaska Native (mean [SD] applied, 0.0007 [0.0002]; matriculated, 0 [0]; *t*_4_ = 6.771; 95% CI, 0.0004 to 0.001; *P* = .001), Black (mean [SD] applied, 0.061 [0.005]; matriculated, 0.039 [0.002]; *t*_4_ = 8.691; 95% CI, 0.014 to 0.027; *P* < .001), Hispanic (mean [SD] applied, 0.099 [0.003]; matriculated, 0.090 [0.0005]; *t*_4_ = 5.476; 95% CI, 0.004 to 0.013; *P* = .003), and Native Hawaiian (mean [SD] applied, 0.0006 [0.0002]; matriculated, 0 [0]; *t*_4_ = 5.874; 95% CI, 0.0003 to 0.001; *P* = .002) applicants as well as applicants who identify with 2 or more races (mean [SD] applied, 0.452 [0.015]; matriculated, 0.508 [0.002]; *t*_4_ = 9.137; 95% CI, −0.074 to −0.039; *P* = .002). White applicants had significantly higher matriculation rates (*P* = .002). Only Asian applicants did not have a statistical difference between the proportions of application and matriculation (Table 2). Comparing 2023 and 2024 data, Asian (χ^2^_1_ = 4.78; *P* = .01), Hispanic (χ^2^_1_ = 3.21; *P* = .04), and White (χ^2^_1_ = 22.273; *P* < .001) applicants to DO programs saw significant increases while the decline among Black applicants was not statistically significant. It is also important to note the absence of individuals who solely identify as American Indian or Alaska Native and Native Hawaiian matriculants in DO programs, underscoring persistent underrepresentation.

**Table 2.** Racial Composition of Doctor of Osteopathic Medicine Applicant Pool and Matriculated Students^a^

Race and Ethnicity	2020		2021		2022		2023		2024	
	Applied	Matriculated	Applied	Matriculated	Applied	Matriculated	Applied	Matriculated	Applied	Matriculated
American Indian or Alaska Native	128 (0.06)	0	244 (0.1)	0	94 (0.04)	0	192 (0.09)	0	187 (0.09)	0
Asian	57 703 (28.5)	2525 (29.1)	69 016 (28.4)	2579 (29.1)	65 220 (30.3)	2632 (29.2)	65 959 (31.5)	2656 (29.0)	63 480 (31.8)	2711 (28.9)
Black or African American	12 013 (5.9)	315 (3.6)	16 917 (7.0)	353 (4.0)	12 668 (5.9)	372 (4.1)	11 985 (5.7)	372 (4.0)	11 954 (6.0)	386 (4.1)
Hispanic, Latino, or of Spanish Origin	19 195 (9.5)	778 (9.0)	25 445 (10.5)	797 (9.0)	21 468 (10.0)	813 (9.0)	20 609 (9.8)	824 (9.0)	19 607 (9.8)	855 (9.1)
Native Hawaiian or Other Pacific Islander	209 (0.1)	0	176 (0.07)	0	168 (0.08)		135 (0.06)	0	62 (0.03)	
White	95 805 (47.3)	4446 (51.2)	110 291 (45.4)	4483 (50.6)	98 807 (45.5)	4565 (50.6)	92 797 (44.2)	4651 (50.8)	86 727 (43.4)	4779 (50.9)
≥2 races	7826 (3.9)	305 (3.5)	9731 (4.0)	324 (3.7)	8963 (4.2)	332 (3.7)	9207 (4.4)	332 (3.6)	8812 (4.4)	345 (3.7)
Unknown race and ethnicity	7266 (3.6)	224 (2.6)	7698 (3.2)	232 (2.6)	6299 (2.9)	232 (2.6)	6182 (2.9)	232 (2.5)	6852 (3.4)	232 (2.5)
URIM, %^b^	19.4	16.1	21.6	16.7	20.1	16.8	20.1	16.6	20.3	16.9
Total, No.^c^	202 651	8676	243 0315	8851	215 493	9029	209 744	9150	199 914	9391

Abbreviation: URIM, underrepresented in medicine.

^a^ Applicant and matriculant data were calculated using data presented by the American Association of Colleges of Osteopathic Medicine Application Service (AACOMAS). AACOMAS presents temporary residents and non-US citizens as a nonresident alien category which is not shown here.

^b^ URIM data presented here were calculated using those who identified as American Indian or Alaska Native; Black or African American; Hispanic, Latino, or of Spanish origin; Native Hawaiian or other Pacific Islander; and those who reported 2 or more races.

^c^ Total data were taken directly from AACOMAS and are greater than the sum of each individual row given that nonresident alien was not included here.

Independent *t* tests revealed widespread disparities when comparing all racial groups with White and Asian applicants. In both MD and DO data, every racial and ethnic group was significantly underrepresented in both applications and matriculation when compared with White individuals. Likewise, compared with Asian individuals, all racial groups except White had significantly lower application and matriculation rates.

When comparing application to MD and DO programs, American Indian or Alaska Native, Black, Hispanic, and White groups were significantly greater among MD programs than DO programs. Asian (mean [SD] DO, 16.16 [9.41]; MD, 0.004 [0.0003]; *t*_4_ = 3.840; 95% CI, 4.474-27.842; *P* = .01) and Native Hawaiian (mean [SD] DO, 0.301 [0.015]; MD, 0.265 [0.018]; *t*_4_ = 3.149; 95% CI, 0.009-0.060; *P* = .01) applicants were significantly more represented in DO programs than in MD programs. In terms of matriculation, all URIM groups had significantly higher matriculation into MD programs compared with DO programs. For Asian individuals, there was no statistically significant difference between MD and DO matriculation. For White individuals, matriculants favored MD programs, but the difference was not statistically significant.

## Discussion

Despite the critical need for a diverse physician workforce, the enrollment of URIM groups remained stagnant over the past 2 decades.^17,18,19^ In response, the Liaison Committee on Medical Education recommended in 2009 the development of pipeline programs to enhance both interest in and access to medical careers for URIM students.^20^ Beyond promoting equity, increasing representation among physicians is beneficial, as patients who share ethnic or gender identities with their doctors experience improved communication, better health care outcomes (including lower newborn mortality and inpatient death rates), and higher patient satisfaction.^6,7,8,9,10^ Additionally, racial concordance between physicians and patients has been linked to greater use of preventive care, reduced emergency department visits, and lower overall health care costs, even when accounting for socioeconomic status.^10,21,22,23^

The findings of this analysis reveal significant shifts in the racial and ethnic composition of matriculating medical school students between the 2023 to 2024 and 2024 to 2025 academic years, coinciding with the recent overturning of affirmative action policies. The largest relative decline was observed in the proportion of American Indian or Alaska Native MD students, which while not statistically significant, decreased from 258 to 201 matriculants, a 22.1% drop. The relative declines in Black or African American and Hispanic students in MD schools (11.6% and 10.8%, respectively) suggest that the removal of race-conscious admissions policies may have disproportionately affected these historically underrepresented groups.

Hispanic is the largest growing ethnic group and makes up approximately 20% of the US population as of the 2023 census, yet it is one of the smallest groups of medical students admitted in the past few years.^15^ Similarly, Black individuals make up 14% of the US population, yet in recent years they are matriculating into MD institutions at decreasing rates, despite steady rates of applications.^15^ In contrast, the percentage of Asian students matriculating into MD schools increased by 8.4%, potentially reflecting a different dynamic in the admissions process after the overturn, though it is important to consider whether this increase is evenly distributed across the diverse subgroups within the Asian population. While DO programs did not see a significant decline in matriculation, DO programs are significantly behind MD programs in matriculating URIM students.

Despite the growing need for physicians throughout the US, there continues to be a lack of equitable matriculation in racial groups that have been historically URIM. While numerous racial groups continue to increase in application to medical school, it is evident from the statistical analysis that all groups have lower matriculation rates than their White and Asian counterparts into both MD and DO classes.

To address these challenges, institutions must explore and invest in alternative strategies that support diversity while complying with new legal constraints. Organizations such as the AAMC and AACOM should continue to advocate for data transparency and support for medical schools navigating this new era. State and federal strategies can also help mitigate declines in diversity, such as increased scholarship support for students from underserved and economically disadvantaged backgrounds, particularly those from rural or medically underserved regions. These race-neutral approaches can expand access while aligning with current legal frameworks. Additionally, investments in minority-serving institutions and targeted pipeline programs remain essential tools in building a more equitable pathway to medical education.

As we prioritize increasing access to care for all populations across the country, notably those who have historically been disenfranchised with poorer outcomes, it is important to create and support policies that encourage diversity. A diverse medical student cohort is essential to producing a physician workforce that reflects the nation’s demographics and can more effectively serve the needs of all communities. As admissions policies shift, institutional commitments to equity, inclusion, and justice must deepen in response.

### Limitations

This study has limitations. We examined publicly available data from AAMC and AACOM. AAMC data used was reported as race and ethnicity responses “alone or in combination,” meaning individuals may be represented in more than 1 racial category. As a result, we were unable to identify or exclude duplicate individuals across categories, and analyses reflect aggregate counts rather than mutually exclusive racial groups. This approach may overrepresent smaller racial groups and limits comparison with datasets that use mutually exclusive classifications. At the same time, reliance on “race alone” categories would exclude individuals identifying with more than 1 race from each respective group, potentially underrepresenting multiracial populations within individual racial and ethnic categories. By using “alone or in combination” data, individuals identifying with multiple racial or ethnic groups are reflected within each relevant category, providing a more inclusive representation of trends across groups. Nonetheless, this approach limits the ability to distinguish single-race from multiracial individuals. Additionally, applicants may elect to not disclose their race, perhaps in fear of limiting their chances of matriculation. The study also does not include other health professions, particularly advanced practice practitioners (APPs) such as nurse practitioners and physician assistants, whose roles are increasingly vital in both inpatient and outpatient settings. Including matriculation data from APP programs could provide a more comprehensive view of the health care education landscape. Finally, applicant pools for MD and DO programs are not mutually exclusive, as many individuals apply to both. This overlap may result in some applicants being counted in both datasets, particularly when assessing trends in applications, acceptances, and matriculations by race or ethnicity. As a result, comparing absolute numbers or interpreting differences between MD and DO pathways should be done cautiously. This may artificially inflate the number of total applicants or obscure meaningful differences in diversity metrics between the 2 application systems.

## Conclusions

The current demographic shifts underscore the far-reaching implications of the Supreme Court’s decision to eliminate race-conscious admissions policies. The changes point to a need for medical schools to explore alternative strategies to promote diversity and inclusion, such as considering socioeconomic factors, geographic diversity, and other holistic admissions practices. Without such measures, medical schools may struggle to maintain equitable representation of underrepresented groups, ultimately further negatively impacting the diversity of the health care workforce and patient care outcomes. For future studies, other timely policy changes could be examined, particularly those regarding hiring pauses. Restrictions to diversity, including the recent ban of diversity, equity, and inclusion programs for institutions receiving government funding, could create additional setbacks and decreased outcomes in both diversity of health care professionals and the quality of patient care. Future studies have the potential to investigate match rates and specialties that students match into based on racial groups, as this gives a realistic picture of the most updated incoming physician workforce.

## Notes

Supplement. Data Sharing Statement
