# Error in Limitations and Table 1 Footnotes

**DOI:** 10.1001/jamanetworkopen.2026.18370

**Published:** 2026-05-22

In the Original Investigation titled “Affirmative Action Repeal and Racial and Ethnic Diversity in US Medical School Admissions,”^1^ published October 10, 2025, there were errors in the Limitations and Table 1 footnotes. The dataset was incorrectly characterized in the Limitations and footnote a of Table 1, which stated that calculations for race and ethnicity were based solely on data from individuals who selected a single race. However, data were in fact derived from race and ethnicity categories reported as “alone or in combination.” The Limitations and Table 1 have been edited to clarify that the same individuals could be represented in more than 1 racial category because of how the data were derived.^1^
