# Supplementary material for: Affirmative Action Repeal and Racial and Ethnic Diversity in US Medical School Admissions
Source: JAMA Netw Open. 2025 Oct 10;8(10):e2535020. doi: 10.1001/jamanetworkopen.2025.35020 (PMC12514628; doi:10.1001/jamanetworkopen.2025.35020)
Supplement: Supplement. — Data Sharing Statement [file jamanetwopen-e2535020-s001.pdf]

Data Sharing Statement

Florescu. Affirmative action repeal and racial and ethnic diversity in US medical school admissions. *JAMA Netw Open.* Pubished October 10, 2025. doi:10.1001/jamanetworkopen.2025.35020

Data Sharing Statement

Data
Data available: No

Additional Information
Explanation for why data not available: The data used in this study are from databases that are publicly available.
